# Does Secondary Inflammatory Breast Cancer Represent Post-Surgical Metastatic Disease?

**DOI:** 10.3390/cancers4010156

**Published:** 2012-02-20

**Authors:** Salman Hashmi, Ladan Zolfaghari, Paul H. Levine

**Affiliations:** Department of Epidemiology and Biostatistics, George Washington University School of Public Services and Health Services, Washington, DC 20037, USA; E-Mails: salmanhashmi79@gmail.com (S.H.); ladan.paul@gmail.com (L.Z.)

**Keywords:** surgery, inflammatory breast cancer, trauma, secondary IBC, dormant micrometastasis, IBC registry, angiogenesis

## Abstract

The phenomenon of accelerated tumor growth following surgery has been observed repeatedly and merits further study. Inflammatory breast carcinoma (IBC) is widely recognized as an extremely aggressive malignancy characterized by micrometastasis at the time of diagnosis, with one interesting subgroup defined as secondary IBC where pathologically identifiable IBC appears after surgical treatment of a primary non-inflammatory breast cancer. One possible mechanism can be related to the stimulation of dormant micrometastasis through local angiogenesis occurring as part of posttraumatic healing. In this report, we review cases of secondary IBC and others where localized trauma was followed by the appearance of IBC at the traumatized site that have been identified by our IBC Registry (IBCR) and hypothesize that angiogenesis appearing as part of the healing process could act as an accelerant to an otherwise latent breast malignancy. It is therefore possible that secondary IBC can be used as a model to support local angiogenesis as an important contributor to the development of an aggressive cancer.

## 1. Introduction

Inflammatory breast cancer (IBC) is widely recognized as an extremely aggressive malignancy that is usually characterized by micro-metastases at the time of diagnosis. IBC is characterized clinically as a rapidly growing tumor with skin manifestation of erythema, warmth and edema and pathologically by invasion of the dermal lymphatics with tumor microemboli. In 1938, Taylor and Meltzer described two types of IBC, the primary form, where the characteristic clinical features were prominent from the outset and the secondary form, where the clinical features appeared subsequent to treatment for a primary non-inflammatory breast cancer [1].

IBC affects approximately 2.5% of women with breast cancer annually in the United States and thus affects more than 4,800 women each year, more than twice as many as those developing chronic myelocytic leukemia or acute lymphocytic leukemia [2]. It is a clinically and pathologically distinct form of breast cancer that is particularly fast growing, highly angiogenic and angioinvasive with its aggressiveness and angiogenicity present from its inception. The precise case definition is controversial [2], with the American Joint Committee on Cancer (AJCC) focusing on a clinical case definition [3], and the Surveillance, Epidemiology and End Results (SEER) Program of the National Cancer Institute focusing primarily on a pathological case definition [4]. While the typical patient presents with pain and a tender, firm enlarged breast with the symptoms developing in less than six months, IBC may be diagnosed with less than half of the breast involved and without the pathological confirmation of dermal lymphatic invasion [2,5]. The skin over the breast is reddened, warm, thickened and often has a pitted appearance termed “peau d’ orange”. The designation “inflammatory” stems from the clinical appearance that mimics an acute inflammation, but this is somewhat of a misnomer [6]. Dermal lymphatic occlusion by tumor infiltrate, a finding which pathologists rely upon to confirm their clinical diagnosis, is believed to lead to increased vascular pressure and stasis, and inflammation does not actually contribute in any consequential way to the skin manifestations [7]. Since IBC tumors produce negligible amounts of most inflammatory cytokines, host inflammatory cells are rarely detected around the tumor stroma [6].

The Inflammatory Breast Cancer Registry (IBCR) was developed to provide a standardized population of IBC patients for epidemiologic and laboratory studies, and among the 156 patients enrolled thus far, eight were identified as having secondary IBC. In our review of these cases and others where localized trauma was followed by the appearance of IBC at the traumatized site, we hypothesized that angiogenesis appearing as part of the healing process could act as an accelerant to an otherwise latent breast malignancy. We present examples of this possible phenomenon which could suggest a population of patients for further investigation.

## 2. Experimental Section

The Inflammatory Breast Cancer Registry (IBCR) was established 1 June 2002 to collect standardized clinical data and biospecimens from patients with IBC in the United States and Canada [2]. Patients with IBC who were entered into the Registry were at least 18 years of age, signed an Informed Consent, agreed to be interviewed and to provide access to medical records and tissue blocks. Patients contacted the Registry after learning about it on the internet or from local oncologists. It was funded initially by the Department of Defense and is now supported by laboratories that use the tissue samples to characterize the disease. In this report we document the histories of two patients with secondary IBC as well as two additional patients whose disease presentation also supports the possible occurrence of IBC secondary to breast trauma. Secondary IBC cases were defined as women who have had surgery for non-inflammatory breast cancer with recurrence manifest as skin erythema shown to be associated with pathologically confirmed tumor emboli in the dermal lymphatics.

### 2.1. Case Reports

#### 2.1.1. IBC 13—Secondary IBC

This 58 year old woman was diagnosed with Stage II infiltrating ductal carcinoma of the right breast in November 1992. She had a modified radical mastectomy and lymph node dissection, three of seventeen nodes being positive for tumor. She was treated with six months of cyclophosphamide, methotrexate and 5-fluorouracil and had no evidence of recurrence. In January 2000 she had a second right breast reconstructive operation and post-operatively redness was noted at the surgical scar site which was first considered to be an allergic reaction and was not biopsied. In August 2000 she developed axillary metastases and she was treated with herceptin, taxotere and carboplatin. While on chemotherapy the redness progressively got worse and was eventually documented as being due to dermal lymphatic invasion. In March 2002 she developed skin involvement of the left breast which also showed dermal lymphatic invasion and she died in May 2008.

#### 2.1.2. IBC 20—Secondary IBC

This 45 year old woman was noted to have a 3.5 cm mass with spiculated margins 13 June 2000. One week later she was diagnosed as having an infiltrating ductal carcinoma and a lumpectomy was subsequently performed. No skin involvement was observed. In July she had a right partial mastectomy and there appeared to be a complete resection with adequate margins beyond the tumor. Three of 14 axillary lymph nodes were noted to be involved with tumor. The surgery was followed by chemotherapy with adriamycin and cytoxan. Erythema of the skin first appeared in September 2000 and biopsy showed dermal lymphatic invasion with tumor microemboli. Despite treatment with Taxol and a right total mastectomy which showed no residual tumor, she developed metastatic disease and died in September 2003.

#### 2.1.3. IBC 36—Post-Traumatic IBC

This 63 year old woman with a history of fibrocystic disease had a routine mammogram in 25 October 2000 which showed “fibrogandular elements” in the left breast. On clinical exam the left breast seemed larger than the right and there was some flattening of the nipple. She had first noticed in August 2000 some tenderness of the left breast and the breast felt engorged. An ultrasound was performed which showed a small cyst in the retroareolar region with dilated retroareolar ducts. The surgeon suggested a ductogram which was performed 18 December 2000 and showed evidence of a filling defect in one of the ducts approximately 5 cm posterior to the areola. The patient described the procedure as being extremely painful and subsequently she had constant pain in the left breast. Subsequently she awoke at night with breast engorgement and heaviness accompanied by nipple inversion and she insisted on an evaluation and biopsy. A biopsy 15 January 2001 showed poorly differentiated ductal carcinoma with extensive lymphatic carcinomatosis. On further evaluation one week later the diagnosis of IBC was made based on erythema and some peau d’ orange under her breast. She received three cycles of adriamycin and cytoxan with an immediate clinical response followed by a left modified radical mastectomy performed on 30 March 2001 which showed a residual mass with multifocal lymphatic involvement, including invasion of the dermal lymhatics. Surgery was followed by a fourth cycle of adriamycin and cyclophosphamide, three cycles of Taxol and radiation therapy which ended 30 August 2001. She was in clinical remission until December 2002 when she developed a significantly elevated CA 15-3 and CEA and a nodule under her clavicle. Biopsy documented metastatic disease and CT scan revealed bilateral pulmonary nodules. Total body scan suggested metastatic disease in the hip. A left breast biopsy showed Her2/neu positive poorly differentiated ductal carcinoma with extensive lymphatic carcinomatosis. She was started on herceptin and taxotere which did not control her disease and she died in May 2003.

#### 2.1.4. IBC 46—Post-Traumatic IBC

This 33 year old woman was in good health and was employed as a civilian working for the military in Guam when she decided to have her nipples pierced. The procedure was performed in early 1999 and subsequently she noticed that the right nipple slowly began to swell. By the end of December the swelling was very prominent and she had the ring removed. On 1 January 2000 she noted a large lump behind the areola. She was able to get an ultrasound in Guam and noticed that the lump had doubled in size in the next five days; she also developed pain with intermittent stinging sensations but no redness. Her original Ob/Gyn doctor in Guam thought it to be an infection and put her on antibiotics. While traveling back to the U.S. where she was hired for a job in Washington, DC, the patient developed erythema of the entire breast with a thumb size port wine stain laterally. She noted that the lump was now of the size of a grapefruit. On evaluation in the U.S. 28 January 2000 the right breast was noted to be tender, painful and swollen with hyperemia, dermal thickening and induration especially in the inferolateral breast. A mammogram that day showed only nonspecific changes suggestive of mastitis. She was treated with antibiotics with no improvement. A biopsy of an indurated part of the port wine stain documented a malignancy and the diagnosis of IBC was made. The mass grew quickly involving more than half the breast with peau d’orange appearance and she received four cycles of adriamycin and cytoxan with excellent response. Mastectomy performed in June 2000 showed infiltrating ductal carcinoma with involved margins and dermal/lymphovascular invasion. All five lymph nodes examined showed metastatic disease. In May 2001 she developed severe back pain and MRI suggested lesions in C7 and T11. She was started on radiation therapy but in June she developed lung lesions and she was treated with taxotere followed by herceptin and letrozole. Her disease persisted but she continued treatment and survived until June 2010.

## 3. Discussion

The phenomenon of accelerated tumor growth following surgery has been observed repeatedly and merits further study in order to determine which patients are more likely to encounter this problem. It appears that there is more than one clinical manifestation of this occurrence, however, and more than one mechanism may be involved. Other reports in this symposium [8,9] have found a hormonal pathogenesis whereby removal of the primary tumor has led to a release of an inhibition on latent metastases. Another possible mechanism, however, is the stimulation of tumor growth through local angiogenesis occurring as part of post-traumatic healing. The central importance of tumor neovascularization has been emphasized by clinical trials using antiangiogenic therapy in breast cancer. Although findings to date have not indicated significant benefits in terms of survival, nevertheless significant improvements in response rates have been documented [10]. “Surgery-driven enhancement of metastases”, the subject of a recent review [11] as well as a focus of this symposium, may well be exemplified by secondary IBC.

IBC is a particularly aggressive form of breast cancer, treated initially with chemotherapy because of the likelihood of dissemination of micro metastases from the outset. The clinical and pathologic findings, while differing in extent from patient to patient, are striking and readily apparent to anyone familiar with this disease. A rapid spread of erythema often with documented invasion of the dermal lymphatics is pathognomonic of IBC. A diagnosis of inflammatory breast carcinoma is made on the basis of the clinical findings. A skin biopsy specimen that is negative for dermal lymphatic invasion does not rule out inflammatory carcinoma [7]. Our experience with the IBC Registry which has currently enrolled 156 well documented patients with the disease, has confirmed that most patients present with the sudden appearance of redness, swelling and tenderness of the breast.

One interesting subgroup of patients, however, are the eight patients in our Registry meeting the case definition of secondary IBC; described by Taylor and Meltzer who noted that “In the group which we would designate as secondary (IBC) the inflammatory manifestations may appear suddenly in a breast which has long been the seat of a scirrhous carcinoma…or it may follow mastectomy for scirrhous carcinoma, either at the original site or the opposite breast” [1]. Our experience with IBC, noted in the case reports above, suggest that local trauma probably mediated in large part by angiogenesis can be an important trigger of IBC. As with primary IBC, the clinical presentation is not uniform, but striking occurrences such as described for IBC patients 13 and 20 clearly link the initial appearance of IBC to the site of surgical trauma.

In this report, we describe two cases compatible with secondary IBC that have been identified by our IBC Registry and two cases where IBC appeared at the site of local trauma. In these four cases, the brief interval between the breast trauma and the appearance of clinical evidence of IBC (Table 1) is understandable in view of the rapidity with which IBC advances. It is reasonable to hypothesize that latent cancer cells remain after surgery and usually do not manifest clinical signs unless stimulated by local angiogenesis. Not only can surgery promote shedding of tumor cells from the malignant tissue into the blood and lymphatic system but it could also eliminate the distant anti-angiogenic effect associated with the primary tumor’s presence (carried by factors such as angiostatin and endostatin) and thus promote the survival of microscopic cancer foci [8]. The tissue damage and subsequent inflammatory response induced by surgery can also lead to elevation of pro-angiogenic factors and growth factors (e.g., EGF) [8]. We propose that consideration be given to focusing on possible parallels between “surgery-driven enhancement of metastases” and secondary IBC to identify opportunities to further understand the mechanism for this phenomenon.

**Table 1:** Date of trauma/ surgery, onset of symptoms, IBC diagnosis and time interval between trauma and surgery and onset of symptoms.

IBC ID	Date of Trauma/Surgery	Onset of Symptoms	Date of IBC Diagnosis	Time Interval (between Trauma/Surgery and IBC Symptoms)
13	January 2000: breast reconstruction	Early 2000 (exact month not specified): redness noted at the surgical scar site; Subsequently contralateral axillary metastasis (August 2000)	August 2000: recurrence of breast cancer, clinically diagnosed as IBC	Approximately 1–3 months
20	July 2000: partial mastectomy	September 2000: erythema noted near the surgical scar site	September 2000: right breast excision performed; biopsy documented dermal lymphatic invasion	2 months
36	December 2000: Ductogram performed	December 2000	January 2001: Biopsy and clinical exam	<1 month
46	January 1999: nipple piercing, Ring removal in December 1999	December 1999	Clinically, December 1999, confirmed on biopsy February 2000	Less than one month between ring removal and first clinical signs

The possibility that trauma can be etiologically related to cancer is raised primarily by a number of case reports. In 1933, Coley and Higinbotham reported 360 cases of bone sarcoma, of which 181 (50%) had histories of trauma, and 205 cases of breast carcinoma of which 70 (34%) were associated with trauma [12]. An impressive series has suggested trauma as a cause of bone cancer [13], and a review of post-surgical bone cancers associated with metal implants identified 22 various bone tumors, 17 since 1980 [14]. Several studies have also observed an increased relative risk of carcinoma associated with a history of nasal trauma or injury [15]. A number of studies have reported brain tumors to be significantly associated with trauma [16] and the role of angiogenesis in the healing process has been suggested as an important contribution to tumor aggressiveness [17,18]. This symposium [8,9] follows previous reports [11,19,20] noting the possible contribution of surgery to aggressive breast cancer, but the major focus in these reports has been the systemic contribution of excising a tumor to promoting the acceleration of distant latent micrometastasis [8,9,20].

Surgery remains an effective therapy for solid tumors in the U.S. and dramatically improves survival rates. Recurrences remain the most important challenge; almost one third of surgical patients will ultimately recur locally and/or systemically [21]. The attribution of a malignancy/metastasis to local trauma by searching for a reason for the disease (recall bias) is always a possibility but in view of the hypothesis that trauma in the form of a surgery can stimulate angiogenesis which can accelerate tumor growth, the documentation of IBC appearing following a surgical event and precipitated by it (Case 13 and 20) merits consideration.

## 4. Conclusions

The evidence presented in this symposium and in careful reviews [8,9,11] indicating that surgery can facilitate the appearance of metastatic disease requires considerable attention. While surgery is clearly an important tool in curing breast cancer and is not questioned in the initial treatment of this disease, perhaps a discussion of the potential risks of surgery in breast reconstruction is warranted, particularly if the patient is apparently disease-free after treatment for IBC.

In view of the hypothesis that trauma can stimulate angiogenesis which can accelerate tumor growth, the documentation of IBC appearing at the site of a traumatic event merits consideration. Our experience with IBC, noted in the case reports above suggest that local trauma probably mediated in large part by angiogenesis can be an important trigger of IBC. Studies on human-murine xenograft models like the Mary-X [22] and WIBC-9 [23] have provided insights on the biology of inflammatory breast cancer. These models can be used to define our hypothesis of surgically induced angiogenesis promoting metastasis at the histological and molecular level. We would therefore suggest that secondary IBC be considered for investigation of one possible mechanism for post-surgical tumor dissemination. A major question is how to identify patients at increased risk for this possible complication. Further attention to animal models and a more systematic study of the risk factors in patients with secondary IBC could be helpful.
